# Can artificial intelligence reduce the interval cancer rate in mammography screening?

**DOI:** 10.1007/s00330-021-07686-3

**Published:** 2021-01-23

**Authors:** Kristina Lång, Solveig Hofvind, Alejandro Rodríguez-Ruiz, Ingvar Andersson

**Affiliations:** 1 Diagnostic Radiology, Department of Translational Medicine, Lund University, Inga Maria Nilssons gata 47, SE-20502 Malmö, Sweden; 2 Unilabs Mammography Unit, Skåne University Hospital, Jan Waldenströms gata 22, SE-20502 Malmö, Sweden; 3 Cancer Registry of Norway, Oslo, Norway; 4 Oslo Metropolitan University, P.O. 5313, Majorstuen, 0304 Oslo, Norway; 5 ScreenPoint Medical BV, Toernooiveld 300, 6525 EC Nijmegen, The Netherlands

**Keywords:** Mammography, Mass screening, Breast cancer, Artificial intelligence

## Abstract

**Objectives:**

To investigate whether artificial intelligence (AI) can reduce interval cancer in mammography screening.

**Materials and methods:**

Preceding screening mammograms of 429 consecutive women diagnosed with interval cancer in Southern Sweden between 2013 and 2017 were analysed with a deep learning–based AI system. The system assigns a risk score from 1 to 10. Two experienced breast radiologists reviewed and classified the cases in consensus as true negative, minimal signs or false negative and assessed whether the AI system correctly localised the cancer. The potential reduction of interval cancer was calculated at different risk score thresholds corresponding to approximately 10%, 4% and 1% recall rates.

**Results:**

A statistically significant correlation between interval cancer classification groups and AI risk score was observed (*p* < .0001). AI scored one in three (143/429) interval cancer with risk score 10, of which 67% (96/143) were either classified as minimal signs or false negative. Of these, 58% (83/143) were correctly located by AI, and could therefore potentially be detected at screening with the aid of AI, resulting in a 19.3% (95% CI 15.9–23.4) reduction of interval cancer. At 4% and 1% recall thresholds, the reduction of interval cancer was 11.2% (95% CI 8.5–14.5) and 4.7% (95% CI 3.0–7.1). The corresponding reduction of interval cancer with grave outcome (women who died or with stage IV disease) at risk score 10 was 23% (8/35; 95% CI 12–39).

**Conclusion:**

The use of AI in screen reading has the potential to reduce the rate of interval cancer without supplementary screening modalities.

**Key Points:**

*• Retrospective study showed that AI detected 19% of interval cancer at the preceding screening exam that in addition showed at least minimal signs of malignancy. Importantly, these were correctly localised by AI, thus obviating supplementary screening modalities*.

• *AI could potentially reduce a proportion of particularly aggressive interval cancers*.

• *There was a correlation between AI risk score and interval cancer classified as true negative, minimal signs or false negative.*

## Introduction

Despite population-based mammography screening and improved and effective treatments, breast cancer is still a major cause of cancer-related death in women. In Europe, 138,000 women were estimated to have died from the disease in 2018 [1]. The aim of screening is to detect the disease in an asymptomatic stage to enable early intervention with improved outcome. However, due to limitations of mammography screening, breast cancer can go undetected. Contributing factors are low sensitivity of mammography in dense breasts, certain cancer growth patterns resulting in subtle mammographic presentation or with a fast growth rate that outpaces screening intervals, as well as radiologists’ reading errors (perceptual or interpretive) [2, 3]. Cancers diagnosed in the interval between two screening rounds, after a negative screening exam, are defined as an interval cancer. Interval cancers usually have less favourable prognosis compared to screen-detected cancer and are more likely to be of higher grade and stage, and with a larger proportion of triple negative and HER2-positive breast cancer [4]. The interval cancer rate is therefore an important indicator on the efficacy of a screening programme [5]. The interval cancer rate in biennial screening is reported to be between 0.8 and 3.0/1000 screened women [2, 6]. In a retrospective review, interval cancers can be classified as either true negative, showing minimal signs or false negative. True negative interval cancers are not visible on the preceding screening mammogram and account for approximately half of all interval cancers [2]. Minimal signs refer to interval cancers with a subtle radiographic appearance at screening that could be regarded as insufficient to recall. False negative interval cancers, on the other hand, could have been recalled in screening but were either missed or misinterpreted by the readers. Depending on the review method, including availability to diagnostic mammograms, it has been shown that up to 30% of all interval cancers are classified as false negatives [2, 6–9], which presents an opportunity for improvement.

Recent development of computer-aided detection (CAD) with artificial intelligence (AI) could provide means to lower the number of missed cancers in mammography screening. Retrospective studies have shown that AI for mammography interpretation can reach human level performance in terms of accuracy [10–14]. AI tools can be used as a decision support for radiologists [15, 16] and as such possibly lower perceptual and interpretive errors, or they can be used as a means to triage exams according to risk of malignancy [17–20]. The potential of using AI in detecting false negative interval cancers, or those with minimal signs, on the preceding screening exams has not yet been investigated.

The purpose of this study was to investigate whether a commercially available AI system for mammography interpretation could detect interval cancer, in particular those retrospectively classified as either false negative or showing minimal signs of malignancy, at screening.

## Materials and methods

### Study population

This retrospective study was approved by the Swedish Ethical Review Authority (ref. 2018/322, 2019-03895). Informed consent was waived by the IRB. Screening mammograms from 461 women consecutively diagnosed with an interval cancer at four different screening sites in Southern Sweden (Malmö, Lund, Helsingborg, Kristianstad) between 2013 and 2017 were included in the study. The Swedish population-based screening programme invites women between age 40 and 74. The screening intervals are 18 and 24 months for women below and over the age of 55, respectively. Double reading is standard procedure.

### Image analysis

Preceding screening mammograms of women included in the study were collected and analysed with an AI system (Transpara v1.5.0, ScreenPoint Medical). The AI system first normalises the intensity of the images to remove variations among vendors. Two different modules based on deep learning convolutional neural networks are applied to the images to detect calcifications and soft tissue lesions [21–23]. Soft tissue and calcification findings are later combined to determine suspicious regional findings. Regional findings are assigned a score of 1–100 and are marked in the images (i.e., CAD-mark) when above a threshold, pre-configured by the user (by default, if higher than 60), while the overall exam is assigned with a malignancy risk score of 1–10 based on the most suspicious finding present across the mammographic views. The malignancy risk scores are calibrated to yield approximately one-tenth of screening mammograms in each category. If, in a screening programme, the threshold for recall is set at risk score 9.01 or over, approximately 10% of the population would be recalled for further investigation. Recall thresholds were also provided by the AI system at risk scores 9.67 and 9.92 corresponding to recall rates of 4% and 1%, respectively.

Published studies, with this and other versions of the AI system, have found that using the above-mentioned functionalities can improve radiologists’ performance when used as a decision support [16] while it could also be used to triage mammograms in screening according to risk score, safely reducing workload in about 20% if exams with score 2 or lower are not read by radiologists [20].

### Interval cancer review

Two breast radiologists with 7 and 47 years of experience (from one of the screening sites) reviewed the preceding mammograms of all interval cancers in consensus and classified them according to interval cancer type: true negative (not visible), minimal signs (retrospectively visible cancer that due to its subtle appearance could not be considered as missed) or false negative (missed or misinterpreted). The review was performed on a dedicated radiology workstation (10-megapixel monitor) in a stepwise approach where the screening exam was reviewed before the diagnostic mammogram to limit hindsight bias. Access to the screen readers’ registered comments (Radiology Information System) and annotations (Picture Archiving and Communication System) were available. Furthermore, they determined if the AI system correctly localised the lesion with a CAD-mark. The review also included a classification of breast density according to Breast Imaging Reporting and Data System (BI-RADS) 5th ed. and the number of women with prior breast surgery (specifically breast reduction surgery), with implants and prevalent screening. Finally, the review included an assessment of women who had died or had metastatic breast cancer (stage IV) as a result of their interval cancer (hereafter referred to interval cancer with grave outcome), based on the clinical history ascertained in the Radiology Information System. The follow-up period after interval cancer diagnosis ranged from 3 to 9 years.

### Statistical analyses

The correlation of interval cancer types in relation to AI risk score was analysed with the Kruskal-Wallis test. Comparison of AI risk scores among different classification groups of interval cancer was performed with a post hoc analysis with the Dunn’s test with Bonferroni correction for multiple comparisons. The potential reduction of interval cancers with AI was determined by the number of interval cancers classified as minimal signs and false negative that were correctly localised by AI, at the different recall rate thresholds. The same conditions were applied in the calculation of the potential reduction of interval cancers with grave outcome. The reductions were computed with 95% confidence intervals (CI) using the Wilson binomial method. The significance threshold was set at 0.05. Open-access statistical packages for Python were used for analyses (www.statsmodels.org/stable/index.html, https://docs.scipy.org/doc/scipy/reference/stats.html).

## Results

### Study population characteristics

Thirty-two women were excluded from the analysis due to import failure (*n* = 3), processing failure due to incompatible modality, e.g. computed radiography (*n* = 27), and diagnosis of lobular carcinoma in situ (*n* = 2). Thus, information from 429 women were included in the analysis. Mean age at screening was 58 years (range 39–76) (Table 1), of which 176 women were under the age of 55, i.e. screened with 18 months interval. Notably, 80% (345/429) of the women had dense breasts (BI-RADS c or d) and 14% (60/429) had undergone breast surgery.

**Table 1:** Characteristics of 429 women diagnosed with interval cancers at four different screening sites in Southern Sweden between 2013 and 2017

	*n* (%)
Prevalent screening	29 (7)
Time from screening to interval cancer	
0–12 months	184 (43)
13–24 months	245 (57)
Prior breast surgery	60 (14)
Breast reduction surgery	17 (4)
Breast implants	8 (2)
BI-RADS breast density	
a	11 (3)
b	73 (17)
c	196 (46)
d	149 (35)

Of the 429 women, 8% (35/429) had an interval cancer with grave outcome. Population characteristics for these women were prevalent screening (*n* = 4), prior breast surgery (*n* = 8, of which 2 had breast reduction surgery), breast implant (*n* = 1) and dense breasts (*n* = 27).

The 429 screening exams had been acquired with the following digital mammography devices: Philips (*n* = 77, 18%), Siemens (*n* = 143, 33%) and General Electric (*n* = 209, 49%).

### Interval cancer classification and AI risk score

**Fig. 1:**
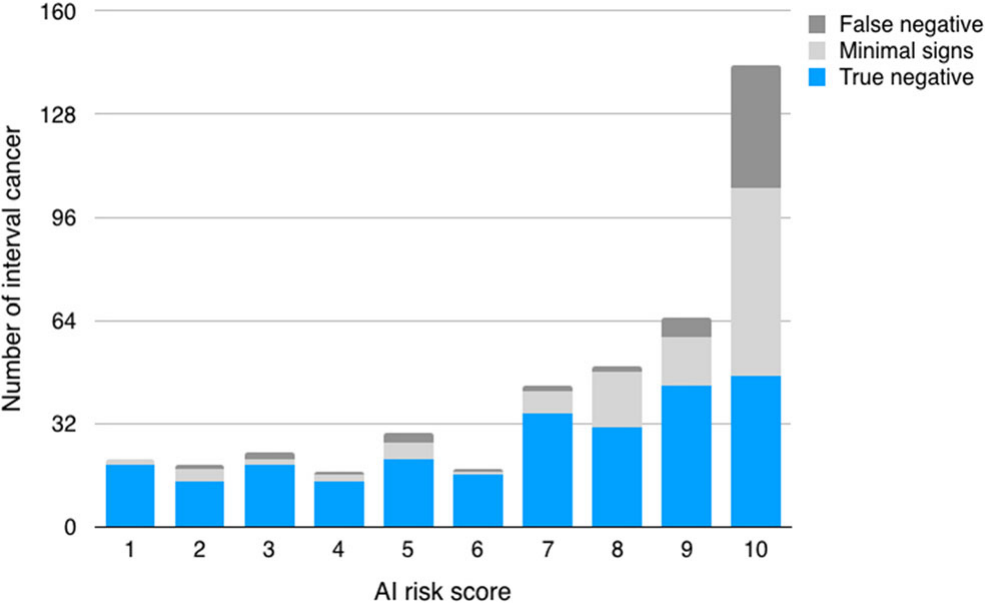
Distribution of interval cancer and classification groups of interval cancer by AI risk score

**Fig. 2:**
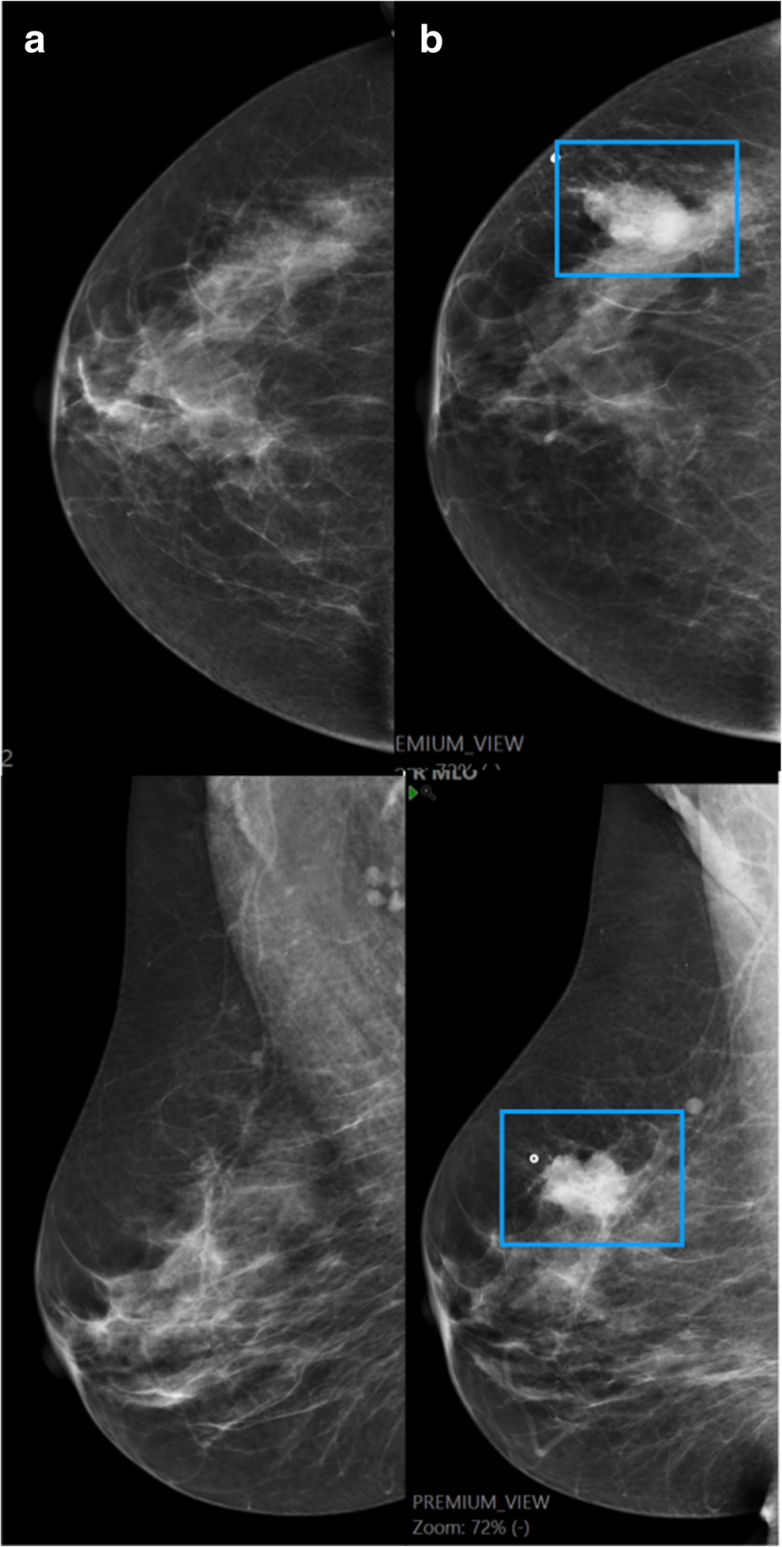
True negative interval cancer. A 56-year-old woman with a negative screen exam. AI assigned a continuous risk score of 8.5 corresponding to exam score 9. The area of the cancer was not CAD-marked (**a**). Sixteen months later, she was diagnosed with a 27-mm-large triple negative breast cancer with histologic grade 3 and Ki67 72% (**b**, blue frame)

The proportion of interval cancers classified as true negative was 60.6% (260/429), while 26.3% (113/429) was classified as minimal signs and 13.1% (56/429) as false negative. Hence, 39.4% (169/429) were considered visible in retrospect, i.e. minimal signs or false negative interval cancers. One in three interval cancers (33.3%, 143/429) had the highest AI risk category of 10 at screening. Of these, 67.1% (96/143) were classified as minimal signs or false negative interval cancer (Fig. 1). The median continuous AI risk scores were 6.7 (IQR 3.8–8.6) for true negative, 9.0 (IQR 7.6–9.6) for minimal signs and 9.7 (IQR 8.2–9.8) for false negative interval cancer, resulting in a statistically significant correlation between classification groups of interval cancer and AI risk score (*p* < .0001). Comparison between interval cancer classification groups showed a significant difference between risk scores for true negative compared with minimal signs and false negatives (*p* < .0001), but no significant difference between minimal signs and false negative interval cancer (*p* = .217). A true negative interval cancer with continuous risk score 8.5 is presented in Fig. 2.

**Fig. 3:**
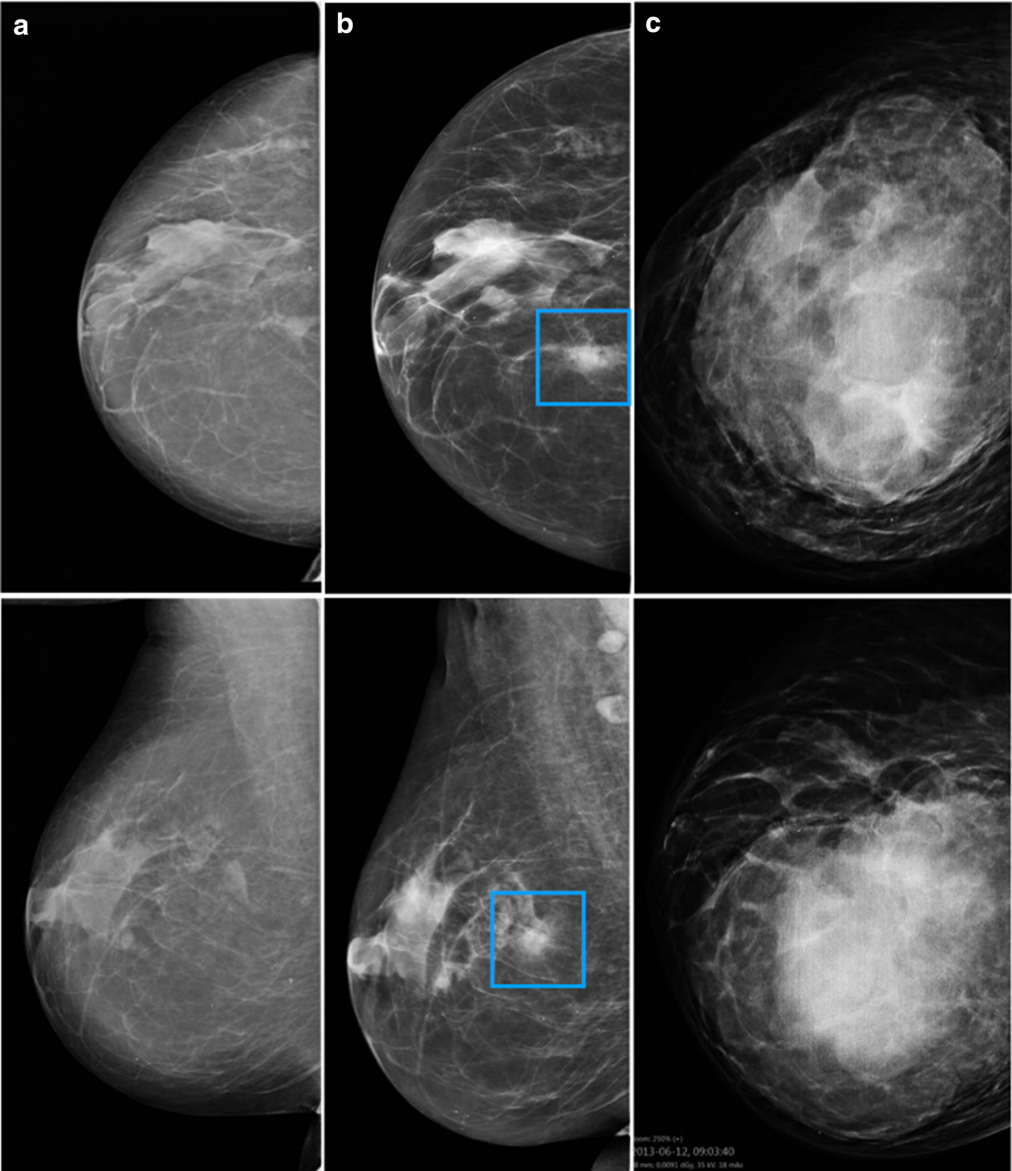
False negative interval cancer. A 57-year-old woman with prior breast reduction surgery undergoing screening classified as negative by double reading at two screening rounds (**a** and **b**). An indistinctly marginated mass, enlarging since the prior screen exam, was correctly identified as high risk by the AI system (exam risk score 10, regional score 81) (**b**, blue frame). Fourteen months later, she was diagnosed with a 12-cm-large metastasised triple negative breast cancer with histologic grade 3 and Ki67 95% (**c**)

The majority of the interval cancers with grave outcome were classified as true negative (57%, 20/35), while 7 were false negative (Fig. 3) and 8 were minimal signs.

### Potential reduction of interval cancer

**Fig. 4:**
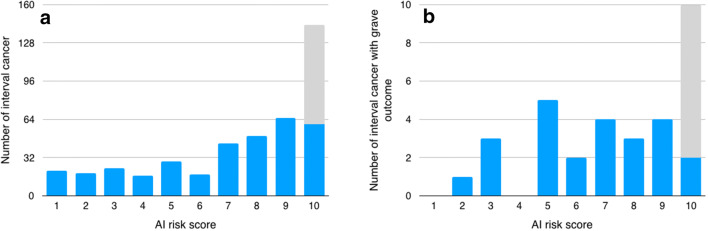
The potential reduction (grey) of interval cancers in screening using AI for all interval cancers (**a**) and for interval cancers with grave outcome (**b**). Note the different scales on the *y*-axis

The total number of interval cancers, specifically those with grave outcome, classified as retrospectively visible, i.e. either minimal signs or false negative, and that were correctly localised by AI for the different AI thresholds is presented in Table 2. Under these premises, the potential reduction of interval cancers in screening for the different AI recall thresholds (AI scores 9.01, 9.67 and 9.92, respectively) was 19.3% (83/429; 95% CI 15.9–23.4), 11.2% (48/429; 95% CI 8.5–14.5) and 4.7% (20/429; 95% CI 3.0–7.1). The maximum potential reduction of interval cancers at AI recall threshold 9.01 (i.e. score 10) is illustrated in Fig. 4a. The corresponding maximum reduction of interval cancers with grave outcome was 8 out of 35; 23% (95% CI 12–39) (Fig. 4b).

**Table 2:** Retrospectively visible interval cancers, i.e. minimal signs or false negative, at different AI risk score thresholds and proportion correctly localised by AI. The thresholds correspond to approx. 10% (score 9.01), 4% (score 9.67) and 1% (score 9.92) recall rates

		Interval cancer classified as minimal signs or false negative	
Recall threshold		*n*, % (95% CI)	Correctly localised, *n*, % (95% CI)
Total (*n* = 169)	9.01	96, 56.8 (49.3–64.3)	83, 49.1 (41.7–56.6)
	9.67	56, 33.1 (26.0–40.2)	48, 28.4 (22.1–35.6)
	9.92	20, 11.8 (7.0–16.7)	20, 11.8 (7.0–16.7)
Interval cancer with grave outcome (*n* = 15)	9.01	9, 60.0 (35.2–84.8)	8, 53.3 (30.1–75.2)
	9.67	5, 33.3 (9.5–57.2)	4, 26.7 (10.9–52.0)
	9.92	3, 20.0 (0.0–40.2)	3, 20.0 (7.0–45.2)

## Discussion

The aim of this retrospective study was to assess the potential of using AI to reduce interval cancers in mammography screening. We found that AI could potentially aid radiologists in detecting up to 19.3% of the interval cancers at screening that in addition showed at least minimal signs of malignancy. Since interval cancers in general are more aggressive than screen-detected cancers, the clinical benefit could be considerable. In this cohort, 8% of the women had interval cancer with grave outcome, of which 23% were correctly located and classified as high risk by AI. Since the shortest follow-up period was 3 years, the number of interval cancers with grave outcome was likely on the lower end.

In a retrospective study on screening data from the USA and UK, McKinney et al showed that a mammography-AI system could reduce false negatives by 9.4% and 2.7% (US and UK dataset, respectively) [10]. In this study, including a larger number of cases, we found a larger reduction of interval cancer. As far as we are aware, no other published study includes an in-depth analysis of AI performance in relation to false negative interval cancers.

The majority (61%) of interval cancers were classified as true negative, of which 82% had dense breasts, a well-known risk factor for interval cancer [2, 24]. Over all, the study population had a high proportion of women with dense breasts, similar to a previously reported interval cancer cohort [25]. Using a screening modality that is less affected by breast density than mammography could be one way of increasing the sensitivity of the screening examination. Breast tomosynthesis can reduce the problem with dense tissue although the results of screening with tomosynthesis in terms of reduction of interval cancer have been conflicting [26, 27]. Supplementary screening with ultrasound and magnetic resonance imaging has been shown to reduce interval cancer rate, but at the expense of false positives and increased cost [28, 29]. This study suggests that AI can be used in a simple way to enhance the sensitivity of mammography screening without introducing supplementary modalities.

We do not suggest that all screening exams with high AI risk should be recalled, which would result in an unacceptable high recall rate (10%). The cancer frequency in mammography screen exams with risk score 10 is about 44/1000 [30], which means that the majority of the exams are cancer-free. In a prior retrospective study on screening data, we found that the highest proportion of false positives were found in risk group 10, which implies that the mammograms were challenging to analyse both for humans and AI [20]. In addition, reader awareness of high AI risk could influence radiologists to lower the threshold to recall, resulting in a reduction of false negatives at the expense of an increase in false positives [3]. To address the potential clinical utility of using AI to lower interval cancer rate at a clinically acceptable specificity, we therefore chose to confine the potential interval cancer reduction to retrospectively visible cancers that were correctly CAD-marked as high risk. Roughly 1/3 of interval cancers received risk score 10, but only half of these were considered to have a suspicious finding that was correctly located with a CAD-mark. It is important to bear in mind that even if a cancer is correctly CAD-marked, it does not necessarily mean that it will be recalled by radiologists, as was shown in a retrospective reader study by Nishikawa et al [31], nor that a cancer necessarily will be diagnosed in the work-up [32, 33], which applies especially to those with minimal signs at screening.

The potential reduction of interval cancer using AI was modest, but involved women diagnosed with interval cancer with grave outcome that most likely would have benefitted from an early detection. Furthermore, even with the use of a high-sensitivity modality such as MRI, not all interval cancers will be detectable at screening [28]. The tumour biology of certain subtypes of breast cancer has a rapid growth rate and/or with an initial subtle or benign radiographic appearance, such as the triple negative subtype [4, 23]. AI performance in relation to tumour biology and stage of interval cancers will be included in future studies.

Notably, the interval cancer cohort in this study included a high proportion of women with prior breast surgery, including surgery of cancer, benign lesions and breast reduction. The surgical deformation of normal breast parenchymal architecture can lead to a tumour masking effect that might compose an independent risk factor of interval cancer. Since we do not have data on how common surgical procedures are in a screening population, a conclusion cannot be drawn. To the best of our knowledge, prior breast surgery has not previously been reported as a risk factor for interval cancer and warrant further studies.

There was a significant correlation between classification groups of interval cancer and AI risk scores. This finding raises an intriguing question whether AI could be used in the clinical audit of interval cancers [24], taking advantage of AI as an interval cancer classifier that is free from hindsight bias. However, this has to be further studied, considering that the review process of interval cancers in this study was subjected to limitations, informed review of a cohort consisting solely of interval cancers. This review method has been shown to lead to a higher proportion of interval cancers classified as false negative compared with a review process that is blinded or with a mix of cases, or even seeded into routine screening [8, 9].

The limitations of this study are several. The informed review process of interval cancer could have inflated the number of false negatives, as mentioned above. The generalizability is further limited due to the use of a single AI system. A study comparing the performance of other AI systems on the same interval cancer cohort is ongoing. In addition, the AI algorithm used in this study has since study completion been updated to an improved version, implying that the potential reduction of interval cancers could be higher. The study was performed in a Swedish screening setting, e.g. starting at a younger age with initial shorter screening intervals than European recommendations [5]. The recall rate, cancer detection rate and interval cancer rate in this screening setting are aligned with European recommendations (approx. 3%, 6/1000 screened women, and 2/1000, respectively). The screening exams were acquired using different mammography devices but did not cover all major mammography vendors. The main limitation is, however, the retrospective design that only provides a theoretical estimation on interval cancer reduction. The use of AI in screening and how the risk scores and CAD-marks influence radiologists’ decisions, and whether AI should be added to double reading or replace one reader, has to be further evaluated in a prospective setting, taking false positives into account.

In conclusion, this study has shown that an AI system detected 19% of interval cancers at the preceding screening mammograms that in addition showed at least minimal signs of malignancy. Importantly, these cancers were correctly located and classified as high risk by AI, thus obviating supplementary screening modalities. AI could therefore potentially aid radiologists in their screen reading to reduce the number of interval cancer and consequently contribute to a further reduction of breast cancer mortality. The implications in a screening programme have to be evaluated in a prospective study.

## Abbreviations

AI: Artificial intelligence
BI-RADS: Breast Imaging Reporting and Data System
CAD: Computer-aided detection
CI: Confidence interval

## References

[1] Ferlay J, Colombet M, Soerjomataram I (2018). Cancer incidence and mortality patterns in Europe: estimates for 40 countries and 25 major cancers in 2018. Eur J Cancer.

[2] Houssami N, Hunter K (2017). The epidemiology, radiology and biological characteristics of interval breast cancers in population mammography screening. NPJ Breast Cancer.

[3] Evans KK, Birdwell RL, Wolfe JM (2013). If you don’t find it often, you often don’t find it: why some cancers are missed in breast cancer screening. PLoS One.

[4] Meshkat B, Prichard RS, Al-Hilli Z (2015). A comparison of clinical-pathological characteristics between symptomatic and interval breast cancer. Breast.

[5] Perry N, Broeders M, de Wolf C, Tornberg S, Holland R, von Karsa L (2008). European guidelines for quality assurance in breast cancer screening and diagnosis. Fourth edition--summary document. Ann Oncol.

[6] Euler-Chelpin MV, Lillholm M, Napolitano G, Vejborg I, Nielsen M, Lynge E (2018). Screening mammography: benefit of double reading by breast density. Breast Cancer Res Treat.

[7] Sankatsing VDV, Fracheboud J, de Munck L (2018). Detection and interval cancer rates during the transition from screen-film to digital mammography in population-based screening. BMC Cancer.

[8] Hofvind S, Skaane P, Vitak B (2005). Influence of review design on percentages of missed interval breast cancers: retrospective study of interval cancers in a population-based screening program. Radiology.

[9] Houssami N, Irwig L, Ciatto S (2006). Radiological surveillance of interval breast cancers in screening programmes. Lancet Oncol.

[10] McKinney SM, Sieniek M, Godbole V (2020). International evaluation of an AI system for breast cancer screening. Nature.

[11] Rodriguez-Ruiz A, Lång K, Gubern-Merida A et al (2019) Stand-alone artificial intelligence for breast cancer detection in mammography: comparison with 101 radiologists. J Natl Cancer Inst. 10.1093/jnci/djy222 10.1093/jnci/djy222 PMC6748773 30834436

[12] Schaffter T, Buist DSM, Lee CI (2020). Evaluation of combined artificial intelligence and radiologist assessment to interpret screening mammograms. JAMA Netw Open.

[13] Wu N, Phang J, Park J (2020). Deep neural networks improve radiologists’ performance in breast cancer screening. IEEE Trans Med Imaging.

[14] Kim H-E, Kim HH, Han B-K (2020). Changes in cancer detection and false-positive recall in mammography using artificial intelligence: a retrospective, multireader study. Lancet Digit Health.

[15] Wu N, Phang J, Park J et al (2019) Deep neural networks improve radiologists’ performance in breast cancer screening. IEEE Trans Med Imaging. 10.1109/TMI.2019.2945514:1-1 10.1109/TMI.2019.2945514 PMC7427471 31603772

[16] Rodríguez-Ruiz A, Krupinski E, Mordang J-J (2018). Detection of breast cancer with mammography: effect of an artificial intelligence support system. Radiology.

[17] Rodriguez-Ruiz A, Lång K, Gubern-Merida A et al (2019) Can we reduce the workload of mammographic screening by automatic identification of normal exams with artificial intelligence? A feasibility study. Eur Radiol. 10.1007/s00330-019-06186-9 10.1007/s00330-019-06186-9 PMC6682851 30993432

[18] Yala A, Schuster T, Miles R, Barzilay R, Lehman C (2019). A deep learning model to triage screening mammograms: a simulation study. Radiology.

[19] Kyono T, Gilbert FJ, van der Schaar M (2020). Improving workflow efficiency for mammography using machine learning. J Am Coll Radiol.

[20] Lång K, Dustler M, Dahlblom V, Åkesson A, Andersson I, Zackrisson S (2020) Identifying normal mammograms in a large screening population using artificial intelligence. Eur Radiol. 10.1007/s00330-020-07165-1 10.1007/s00330-020-07165-1 PMC7880910 32876835

[21] Mordang J-J, Janssen T, Bria A, Kooi T, Gubern-Mérida A, Karssemeijer N, Tingberg A, Lång K, Timberg P (2016). Automatic microcalcification detection in multi-vendor mammography using convolutional neural networks. Breast imaging.

[22] Bria A, Karssemeijer N, Tortorella F (2014). Learning from unbalanced data: a cascade-based approach for detecting clustered microcalcifications. Med Image Anal.

[23] Kooi T, Litjens G, van Ginneken B (2017). Large scale deep learning for computer aided detection of mammographic lesions. Med Image Anal.

[24] Cornford E, Sharma N (2019). Interval cancers and duty of candour, a UK perspective. Curr Breast Cancer Reports.

[25] Ciatto S, Visioli C, Paci E, Zappa M (2004). Breast density as a determinant of interval cancer at mammographic screening. Br J Cancer.

[26] Hovda T, Holen ÅS, Lång K (2019). Interval and consecutive round breast cancer after digital breast tomosynthesis and synthetic 2D mammography versus standard 2D digital mammography in BreastScreen Norway. Radiology.

[27] Houssami N, Bernardi D, Caumo F (2018). Interval breast cancers in the ‘screening with tomosynthesis or standard mammography’ (STORM) population-based trial. Breast.

[28] Bakker MF, de Lange SV, Pijnappel RM (2019). Supplemental MRI screening for women with extremely dense breast tissue. N Engl J Med.

[29] Ohuchi N, Suzuki A, Sobue T (2016). Sensitivity and specificity of mammography and adjunctive ultrasonography to screen for breast cancer in the Japan Strategic Anti-cancer Randomized Trial (J-START): a randomised controlled trial. Lancet.

[30] Christiana B, Alejandro R-R, Christoph M, Nico K, Sylvia HH-K (2020) Going from double to single reading for screening exams labeled as likely normal by AI: what is the impact?, Proc. SPIE 11513, 15th International Workshop on Breast Imaging (IWBI2020) 115130D. 10.1117/12.2564179

[31] Nishikawa RM, Schmidt RA, Linver MN, Edwards AV, Papaioannou J, Stull MA (2012). Clinically missed cancer: how effectively can radiologists use computer-aided detection?. AJR Am J Roentgenol.

[32] Ciatto S, Houssami N, Ambrogetti D, Bonardi R, Collini G, Del Turco MR (2007). Minority report - false negative breast assessment in women recalled for suspicious screening mammography: imaging and pathological features, and associated delay in diagnosis. Breast Cancer Res Treat.

[33] Lameijer JRC, Voogd AC, Pijnappel RM (2020). Delayed breast cancer diagnosis after repeated recall at biennial screening mammography: an observational follow-up study from the Netherlands. Br J Cancer.

